# Premyogenic progenitors derived from human pluripotent stem cells expand in floating culture and differentiate into transplantable myogenic progenitors

**DOI:** 10.1038/s41598-018-24959-y

**Published:** 2018-04-26

**Authors:** Fusako Sakai-Takemura, Asako Narita, Satoru Masuda, Toshifumi Wakamatsu, Nobuharu Watanabe, Takashi Nishiyama, Ken’ichiro Nogami, Matthias Blanc, Shin’ichi Takeda, Yuko Miyagoe-Suzuki

**Affiliations:** Department of Molecular Therapy, National Institute of Neuroscience, National Center of Neurology and Psychiatry, Tokyo, 187-8502 Japan

## Abstract

Human induced pluripotent stem cells (hiPSCs) are a potential source for cell therapy of Duchenne muscular dystrophy. To reliably obtain skeletal muscle progenitors from hiPSCs, we treated hiPS cells with a Wnt activator, CHIR-99021 and a BMP receptor inhibitor, LDN-193189, and then induced skeletal muscle cells using a previously reported sphere-based culture. This protocol greatly improved sphere formation efficiency and stably induced the differentiation of myogenic cells from hiPS cells generated from both healthy donors and a patient with congenital myasthenic syndrome. hiPSC-derived myogenic progenitors were enriched in the CD57(−) CD108(−) CD271(+) ERBB3(+) cell fraction, and their differentiation was greatly promoted by TGF-β inhibitors. TGF-β inhibitors down-regulated the *NFIX* transcription factor, and *NFIX* short hairpin RNA (shRNA) improved the differentiation of iPS cell-derived myogenic progenitors. These results suggest that *NFIX* inhibited differentiation of myogenic progenitors. hiPSC-derived myogenic cells differentiated into myofibers in muscles of *NSG-mdx*^*4Cv*^ mice after direct transplantation. Our results indicate that our new muscle induction protocol is useful for cell therapy of muscular dystrophies.

## Introduction

Currently, there is no satisfactory therapy for Duchenne muscular dystrophy (DMD). Myoblast transplantation is one of the promising therapeutic strategies because wild-type mouse myoblasts have been shown to fuse with host dystrophic myofibers and express dystrophin at the sarcolemma in a DMD model, the *mdx* mouse^1^. However, myoblast transfer therapy performed in the early 1990s did not improve muscle function in DMD patients^2,3^. The scarcity of muscle satellite cells, which are activated after isolation and proliferate to become myoblasts in muscle, is one of the factors that limit the use of cell therapy because culturing myoblasts lowers their regenerative capacity^4,5^. In contrast, human induced pluripotent stem cells (hiPSCs) can be induced to differentiate into various cell types, including skeletal muscle, even after extensive expansion *in vitro*^6^. Therefore, hiPS cells are expected to be an inexhaustible source of myogenic stem cells for cell therapy.

Many protocols have been reported to induce skeletal muscle cells from human embryonic stem (ES) cells or hiPS cells^7^. The majority rely on induction of exogenous expression of *MyoD* or *Pax7* genes in hiPS cells by using mRNA^8^, lentiviral vectors^9–11^, adenoviral vectors^12^, or transposon vectors^13^. These methods are powerful for induction of skeletal muscle cells, but transgene-mediated muscle induction is not suitable for cell therapy. In contrast, a relatively few reports describe successful induction of myogenic stem cells and progenitor cells without forced expression of *MyoD*, *Pax7*, or other myogenic factors^14–20^. The EZ sphere culture method, which is the simplest among them^21,22^, employs free-floating spherical cultures of human ES cells or hiPS cells in a flask with an ultra-low cell attachment surface in the presence of high levels of fibroblast growth factor-2 (FGF-2) and epidermal growth factor (EGF) for six weeks. This method is automatable and scalable to produce the clinically required numbers of cells. However, the efficiency of induction varies considerably among hPSC lines^21,22^.

In this study, to improve induction efficiency, we induced dermomyotomal progenitors stepwise from hiPS cells using a recently reported protocol^18,19^, and then cultured them as floating spheres^21,22^. Chemical induction of these progenitors improved the efficiency of sphere formation at the beginning of the floating culture and supported robust growth of myogenic spheres derived from both normal and disease-specific iPSCs. Induced myogenic progenitors efficiently differentiated into multinucleated myotubes in the presence of TGF-β inhibitors. Gene expression analysis and knockdown experiments suggested that TGF-β inhibits differentiation of myogenic progenitors partly via the *NFIX* transcription factor. We also show that hiPSC-derived myogenic cells transplanted into immune-deficient *mdx* mice differentiated into myofibers and expressed dystrophin. Our results suggest that our new sphere method is useful for hiPSC-based cell therapy of muscle.

## Results

### Continuously stirred floating culture scaled up derivation of myogenic cells from human iPS cells

To obtain sufficient numbers of myogenic cells for cell therapy, we first combined the EZ sphere method^21^ with a continuously stirred floating culture system using a bioreactor (Supplementary Figure 1A). As expected, the cell yield was increased (average 5.8-fold, maximum 16.4-fold) by continuous low-speed stirring (Supplementary Figure 1B), but there was no increase in the percentage of myogenic spheres by the stirred suspension culture compared to the original method (Supplementary Figure 1D). In addition, the four iPS cells (253G4, 201B7, 454E2, and 409B2) formed multinucleated myotubes with quite different efficiencies (Supplementary Figure 1C).

### Reproducible induction of premyogenic progenitors from human iPS cells using CHIR-99021 and LDN-193189

**Figure 1:**
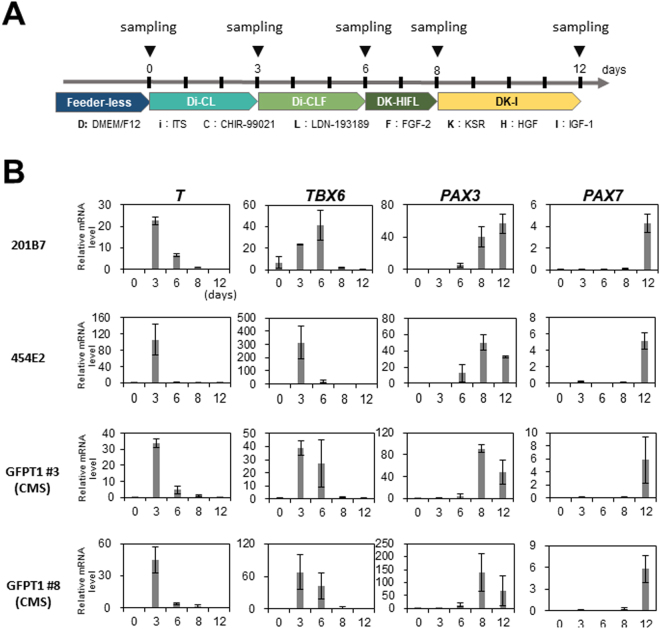
Stepwise derivation of premyogenic progenitors by CHIR-99021 and LDN-193189. (**A**) Initial four steps of the myogenic differentiation protocol for human iPSCs^19^. Three, 6, 8, and 12 days after starting the induction (▼), cells were collected, and total RNA was extracted for RT-qPCR. D: DMEM/F12, i: ITS, C: CHIR-99021, L: LDN-193189, F: FGF-2, K: KSR, H: HGF, and I: IGF-1. The detailed composition of the medium was described in ref.^19^. (**B**) RT-qPCR analysis of *T*, *TBX6*, *PAX3*, and *PAX7* expression in 201B7, 454E2, GFPT1 #3, and GFPT1 #8 iPS cell lines at different time points. Data are from three independent experiments. CMS: congenital myasthenic syndrome.

For efficient induction of myogenic cells, we thought that induction of the paraxial mesoderm was the most critical step. Therefore, we investigated whether the dual modulation of Wnt and BMP pathways using CHIR-99021 and LDN-193189, recently reported by Chal *et al*., increases the efficiency of sphere-based muscle induction. First, we examined whether the reported method correctly induces expression of the genes that are expressed in the paraxial mesoderm. For this analysis, we used two normal hiPSC lines (201B7 and 454E2) and two hiPSC lines derived from a patient with congenital myasthenic syndrome (CMS) due to a *GFPT1* mutation (GFPT1 #3 and GFPT1 #8). *T (BRACHYURY)* was transiently expressed in Di-CL medium. *TBX6* was induced in Di-CL medium and downregulated in DK-HIFL medium. *PAX3* expression was induced in DK-HIFL medium. Finally, *PAX7* was induced in all iPSC clones cultured in DK-I medium with good reproducibility (Fig. 1).

### Premyogenic progenitors efficiently differentiated into myogenic cells in floating culture

**Figure 2:**
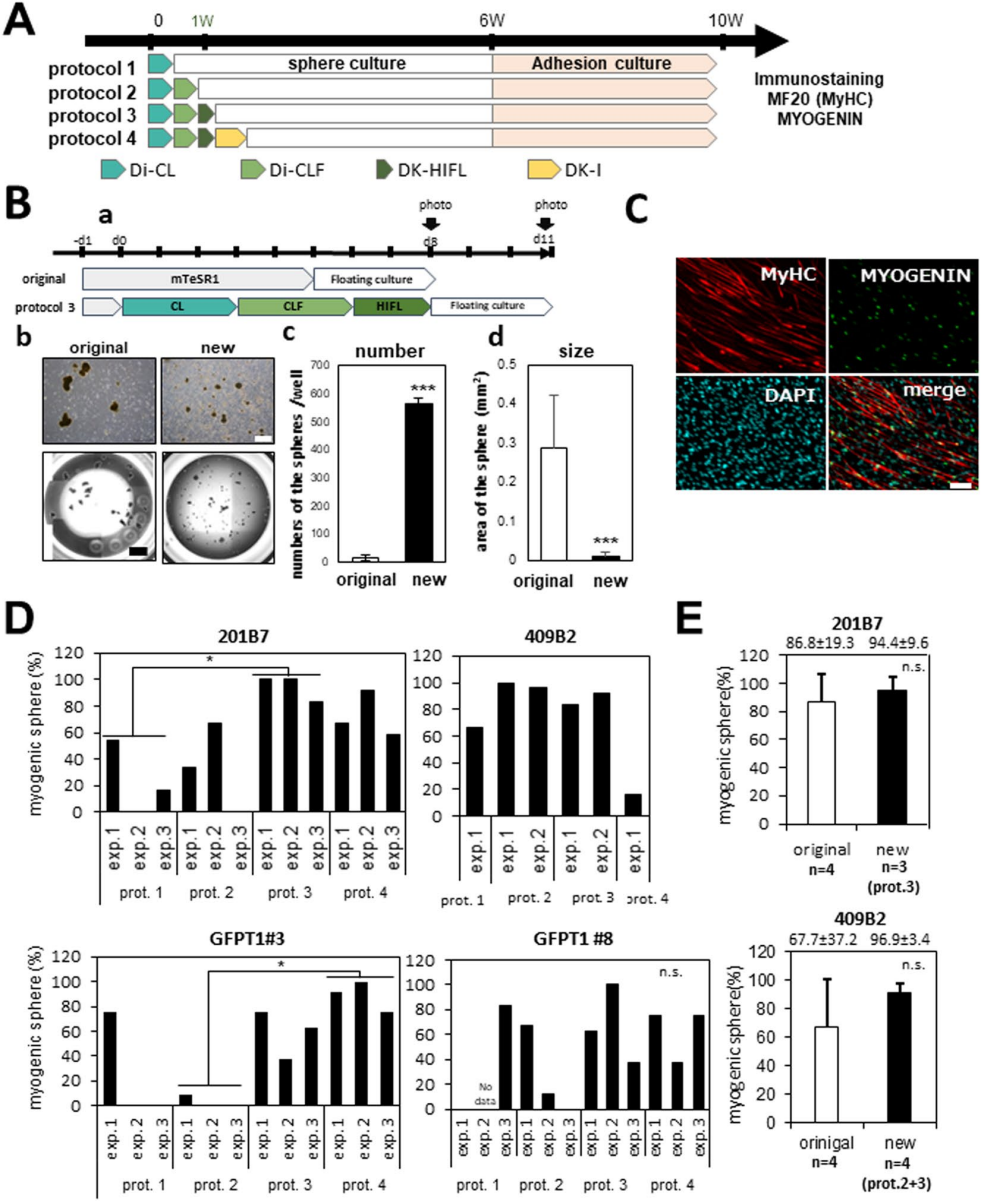
Premyogenic progenitors induced by combination of CHIR-99021 and LDN-193189 were successfully expanded in floating culture and differentiated into skeletal muscle lineage. (**A**) Experimental design. Four different protocols (protocol 1–4) of induction were tested for its efficiency using four iPS cell lines (201B7, 409B2, GFPT1 #3, and GFPT1 #8). After treatment with CHIR-99021 and LDN-193189, cells were cultured as spheres as described^21^. Each sphere was then plated onto collagen-coated 24-well plates at one sphere per well and cultured in 10% FBS/DMEM for 4 weeks. (**B**) 201B7-Myf5-tdTomato cells, a subline of 201B7, were plated onto iMatrix-coated 6-well plates at 2.2 × 10^5^ cells/well. Half of them were cultured in mTeSR1 for 6 days. Another half were cultured in CL for 3 days, CLF for 3 days, and HIFL for 2 days. Then both groups were transferred to floating culture, and 3 days later, the numbers of spheres b and the diameter of all spheres (**C**) were measured using ImageJ. n = 3 per group. t-test. ***P < 0.001. Scale bars in (b) indicate 1 mm (white) or 2 mm (black). (**C**) Representative images of MHC (MF20) (red) and MYOGENIN (green) immunostaining of muscle differentiation of 201B7. Scale bar = 200 μm. (**D**) The percentage of myogenic spheres derived from 201B7 (3 experiments), 409B2 (4 experiments), and GFPT1-iPSCs (#3 and #8)(3 experiments for each) using four different protocols shown in A. (**E**) Comparison of the percentages of myogenic spheres of 201B7 or 409B2 cultured in the EZ sphere method (original) vs. the new method (new). Average ± SD.

After CHIR-99021 and LDN-193189 treatment, we collected the differentiating cells using a cell scraper, transferred them to a floating culture at four different time points (protocols 1–4 in Fig. 2A), and cultured them as floating spheres as described by Hosoyama *et al*.^21^. Then, to evaluate the myogenic activity, we plated one sphere per well in 10% FBS/DMEM medium on collagen-coated 24-well plates. After 4 weeks adhesion culture, the cells were stained for muscle myosin heavy chain (MF20) and MYOGENIN, which is expressed in differentiating myoblasts and myotubes (Fig. 2). Importantly, CHIR-99021 and LDN-193189 treatment increased the numbers of spheres formed compared with the original EZ sphere method. In contrast, the original method gave rise to much fewer and bigger spheres with irregular shapes (Fig. 2B). The highest percentage of myogenic spheres was obtained using protocol 3 or 4, although 409B2 cells efficiently differentiated into myogenic cells using protocol 2 or 3 (Fig. 2B). The average muscle induction efficiency of the new protocol was higher than those of original EZ sphere methods, although there was no statistically significant difference due to the large variation in the inductions using the original method (Fig. 2E, Supplementary Table 1**)**. Floating culture over six weeks reduced the myogenic activity (Supplementary Figure 3). This result is consistent with the results of the original EZ sphere method^21^.

### Gene expressions of myotubes induced by sphere culture

To determine the properties of hiPSC-derived muscle cells, we examined the expression of regulators of myogenesis (*PAX3*, *PAX7*, *MYF5*, *MRF4*, *MYOD*, *SIX1*, *EYA4*, *KBTBD10*, *MYOGENIN*) and muscle proteins (*MYL1*, *TNNC2*, *MYH1*, *MYH2*, *MYH3*, *DYSTROPHIN*, *N-CAM*, *M-CADHERIN*) in myotubes by RT-qPCR. hiPSC-derived myotubes induced by the sphere method were similar to human iPSC-derived, MyoD-induced myotubes in expression of myogenic genes (Supplementary Figure 2). Human iPSC-derived myotubes showed lower expressions of myogenic transcription factors (*PAX7*, *MYOD*, *MYF5*, *MRF4*) and sarcomeric genes (*MYL1*, *TNNC2*, *MYH2*, *MYH3*, *MYH11*) than myoblasts formed by adult myoblasts (Supplementary Figure 2). The expression of embryonic myosin heavy chain (*MYH3*) was higher, but the expressions of adult type MHC IIx (*MYH1*) and adult type MHC IIa (*MYH2*) were much lower in myotubes formed *in vitro* than in adult skeletal muscle (Supplementary Figure 2).

### Sorting myogenic cells using cell surface markers

**Figure 3:**
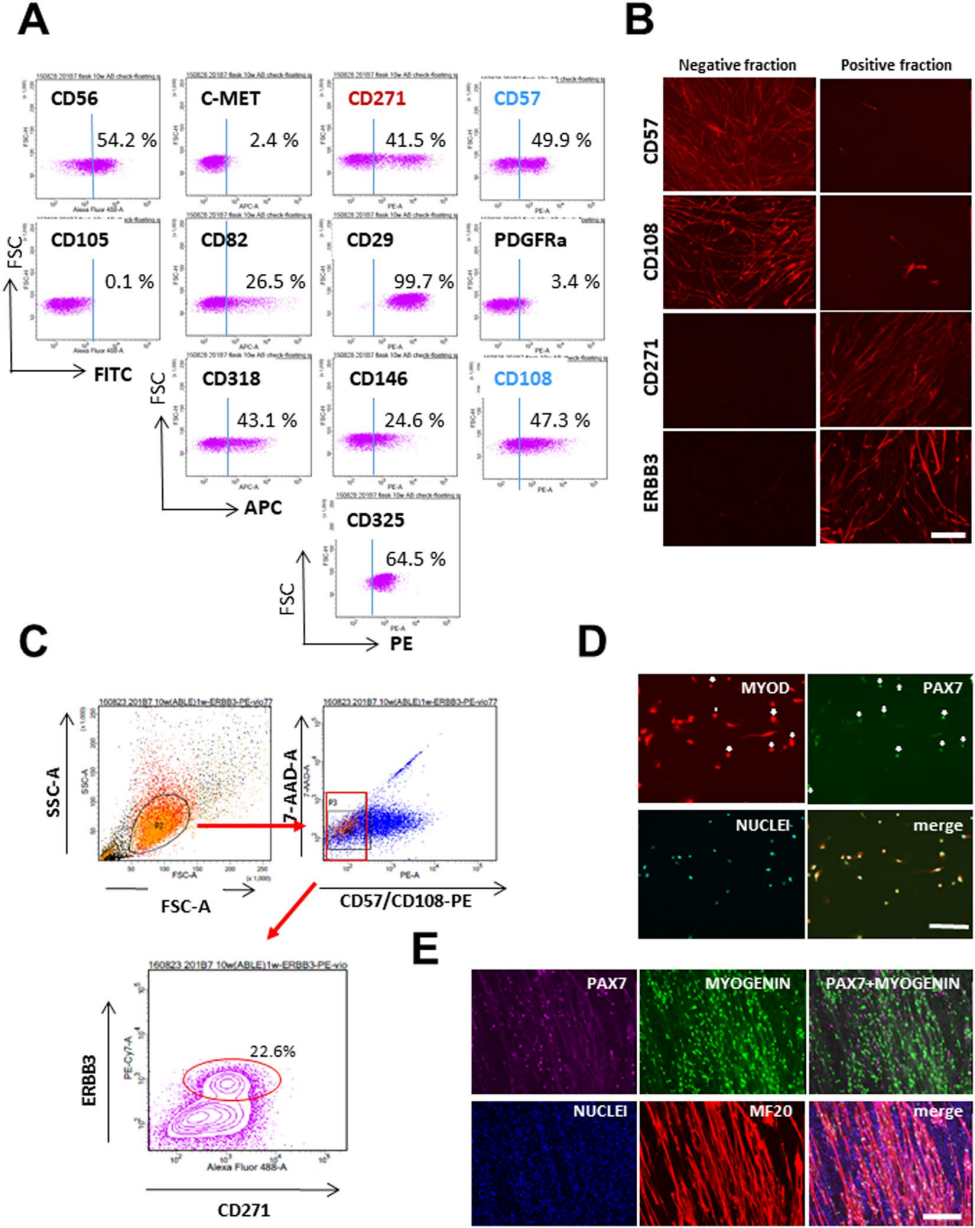
hiPSC-derived myogenic cells are enriched in CD57-negative, CD108-negative, CD271-positive, and ERBB3-positive cell fraction. (**A**) After 6-week-EZ-sphere culture, human iPSC (201B7)-derived sphere cells were cultured on collagen-coated dishes in 10%FBS/DMEM for 4 weeks. The cells were then dissociated into single cells and analyzed by FACS. A representative FACS analysis using 12 Abs is shown with the percentages of positive fractions. (**B**) hiPS cells (253G4) were cultured in a bioreactor for 10 weeks, and then plated onto collagen-coated 100-mm dishes and cultured in 10% FBS/DMEM for one week. The cells were then FACS-sorted using CD57, CD108, CD271, or ERBB3, and each fraction was plated onto 24-well plates and cultured again in 10% FBS/DMEM. Note that MF20-positive (red) myotubes were exclusively found in the CD57-negative, CD108-negative, CD271-positive, or ERBB3-positive fractions. (**C**) After 6-week sphere culture and subsequent 4-week adhesive culture on collagen-coated 10 mm plates, cells were analyzed using FACS. (**D**) Forty-eight hours after sorting, cells were stained for PAX7, MyoD, and nuclei (Hoechst). MyoD was positive in more than 95% cells, while *PAX7* was expressed in 30–40% of the sorted cells (n = 3). (**E**) CD57(−) CD108(−) CD271(+) ERBB3(+) cells were cultured in 10% FBS/DMEM for one week, and immunostained for PAX7(magenta), MYOGENIN(red), MYOSIN HEAVY CHAIN (MF20, violet), and nuclei (Hoechst33258, blue). Many myotubes with clustered nuclei were formed. *PAX7* was expressed in nuclei of mononuclear cells. Scale bars in B, D, E indicate 200 μm.

To analyze the properties of myogenic progenitors, we examined cell surface markers on sphere cells derived from the four hiPSC clones (201B7, 253G4, 409B2, and 454E2) at various time points using more than 20 antibodies (data not shown). After suspension culture, all cells were negative for TRA-1-60, TRA-1-81, and SSEA4, suggesting that no undifferentiated iPS cells remained in the culture (data not shown). When examined after six-week sphere culture and four-week adhesion culture, CD271, which was expressed on postnatal myoblasts but not fibroblasts in our preliminary FACS screening for candidates of cell surface markers (data not shown), was expressed on more than 60% of iPSC-derived sphere cells (Fig. 3). After cell sorting, myotubes were formed exclusively by the CD271-positive fraction, but many CD271-positive fraction cells were non-myogenic, indicating that CD271 is not sufficient for purification of myogenic progenitors. ERBB3, which has been recently demonstrated to be an excellent cell surface marker on PAX7+ muscle progenitor cells derived from hPSCs using two muscle induction protocols^20^, was expressed on a small fraction of sphere cells. Myotubes were formed exclusively by the ERBB3-positive fraction. Previously, we reported that the combination of CD56 (NCAM) and CD82 effectively enriched hiPSC-derived myogenic cells^23^, but ERBB3 enriched myogenic cells much more powerfully than the combination of CD82 and CD56. Sphere cells contained both CD57-positive and -negative cells. MF20-positive myotubes were formed exclusively by CD57-negative cells (Fig. 3B). Screening of surface markers of adult myoblasts using BD Lyoplate™ Screening Panels revealed that *CD108* was expressed on both fibroblasts and myoblasts from adult skeletal muscle (Nishiyama *et al*., unpublished observation). Nonetheless, hiPSC-derived myogenic cells were highly enriched in the CD108-negative fraction (Fig. 3B). At present we have no explanation for this discrepancy. *CD108* might be up-regulated in myogenic cells postnatally. A commercial M-cadherin monoclonal antibody recognized myogenic cells after non-enzymatic dissociation by Gibco^®^ Cell Dissociation buffer but did not recognize the molecule after trypsin treatment, making it difficult to isolate myogenic progenitors from tightly packed spheres (data not shown). Fractionation with CD146/MCAM or CD318 failed to enrich myogenic cells (data not shown). We next sorted CD57(−) CD108(−) CD271(+) ERBB3(+) cells (Fig. 3C), and examined their *PAX7*, *MYOD*, *MYOGENIN*, and *NFIX* expressions by immunocytostaining (Fig. 3D,E). *MYOD* was expressed in most of the cells. *PAX7* was expressed in 30–40% of the sorted cells when fixed within 48 hours after FACS sorting (Fig. 3D). After plating onto collagen-coated dishes in 10% FBS/DMEM, hiPSC-derived CD57(−) CD108(−) CD271(+) ERBB3(+) cells fused to form multinucleated myotubes. *MYOGENIN* was expressed mainly in multinucleated myotubes (Fig. 3E). In contrast, *PAX7* was expressed in mononuclear cells between myotubes (Fig. 3E).

### TGF-β inhibition promoted differentiation of hiPSC-derived myogenic progenitors *in vitro*

**Figure 4:**
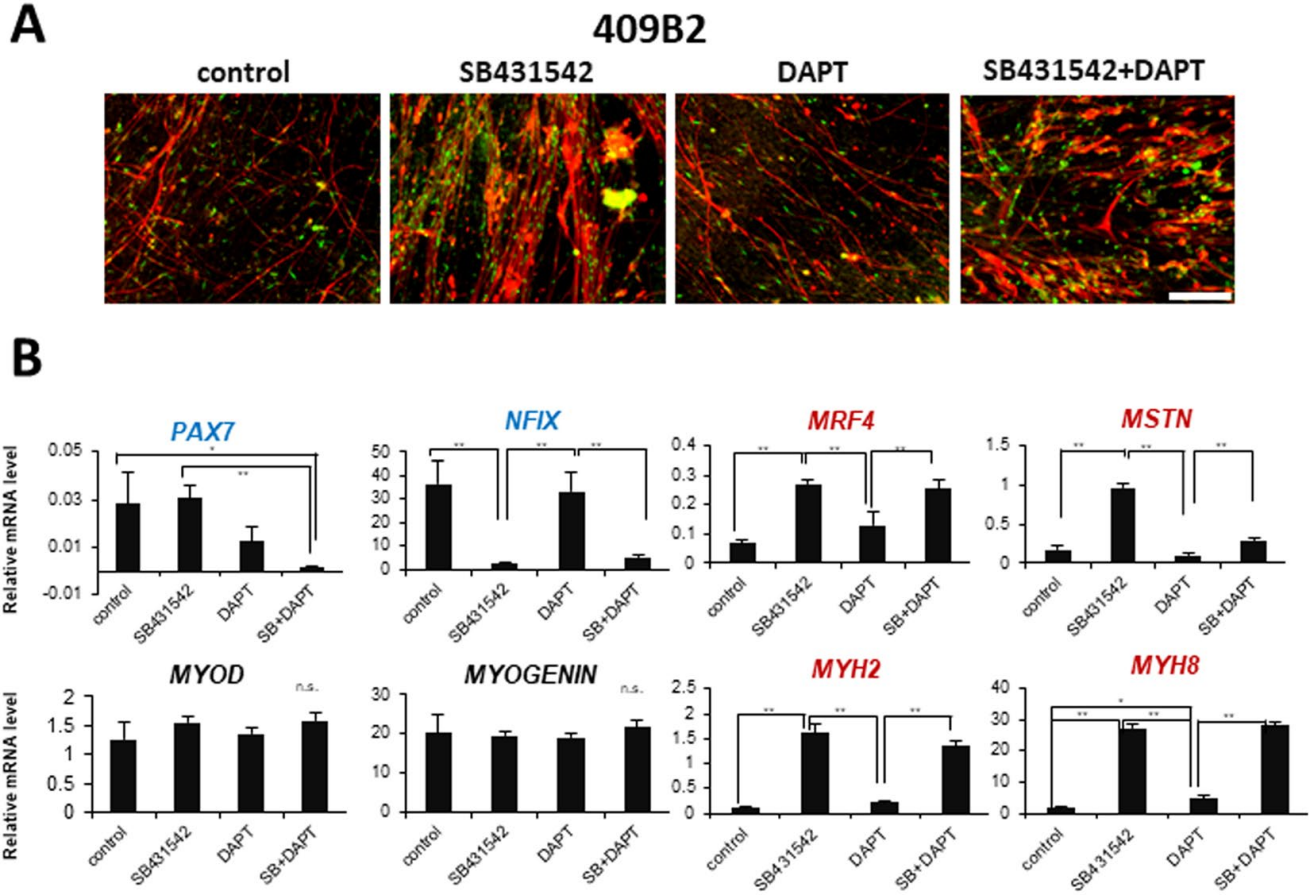
Effects of SB431452 and DAPT1 on differentiation of hiPSC-derived myogenic cells. (**A**) After 6-week muscle induction in sphere culture and 4-week adhesion culture, 409B2 iPS cells were cultured in the presence of SB431542 (TGF-β inhibitor), DAPT (Notch inhibitor), or both (SB431542 + DAPT). Ten days after administration, muscle differentiation was evaluated with MF20 (red), and MYOGENIN (green). Scale bar = 100 μm. (**B**) RT-qPCR analysis of myogenic regulators and muscle-specific genes in control, SB431542-, DAPT-, and SB431542 + DAPT-treated myogenic progenitors derived from 409B2 iPSCs. **p < 0.01, *p < 0.05, n.s. (n = 3).

Mouse embryonic and fetal myoblasts respond differently to TGF-β^24^. We examined whether TGF-β inhibition promotes muscle differentiation of hiPSC-derived myogenic progenitors to determine the developmental stage. A TGF-β receptor type I kinase inhibitor SB431542 (Fig. 4, Supplementary Figure 4) or A83-01 (data not shown) dramatically improved myogenic differentiation of iPSC-derived myogenic progenitors. This result is consistent with the results recently published by Hicks *et al*.^20^, suggesting that our cells have the phenotype of fetal myoblasts, and probably equivalent to their hiPSC-skeletal muscle progenitor cells (SMPCs). RT-qPCR analysis revealed that *MRF4*, *MSTN*, *MYH2(fast IIa)*, and *MYH8 (fetal/perinatal MHC)* were up-regulated by SB431542. Interestingly, SB431542 reduced the expression of *NFIX*, but unexpectedly did not increase mRNA levels of *MYOD* or *MYOGENIN*. Although Notch signal is reported to inhibit terminal differentiation of muscle stem cells in mice^25^, a Notch inhibitor, DAPT (γ-secretase inhibitor), showed no significant effect on muscle differentiation of hiPSC-derived muscle progenitors. Interestingly, the expression of PAX7 was not down-regulated by the treatment of SB431542, but was significantly reduced by dual treatment of SB431542 and DAPT (Fig. 4, Supplementary Figure 4). Addition of an ERBB3 ligand, a recombinant neuregulin-beta1, showed no significant effects on differentiation of iPSC-derived myogenic progenitors (data not shown).

### *NFIX* maintained undifferentiated state of hiPSC-derived myogenic progenitors

**Figure 5:**
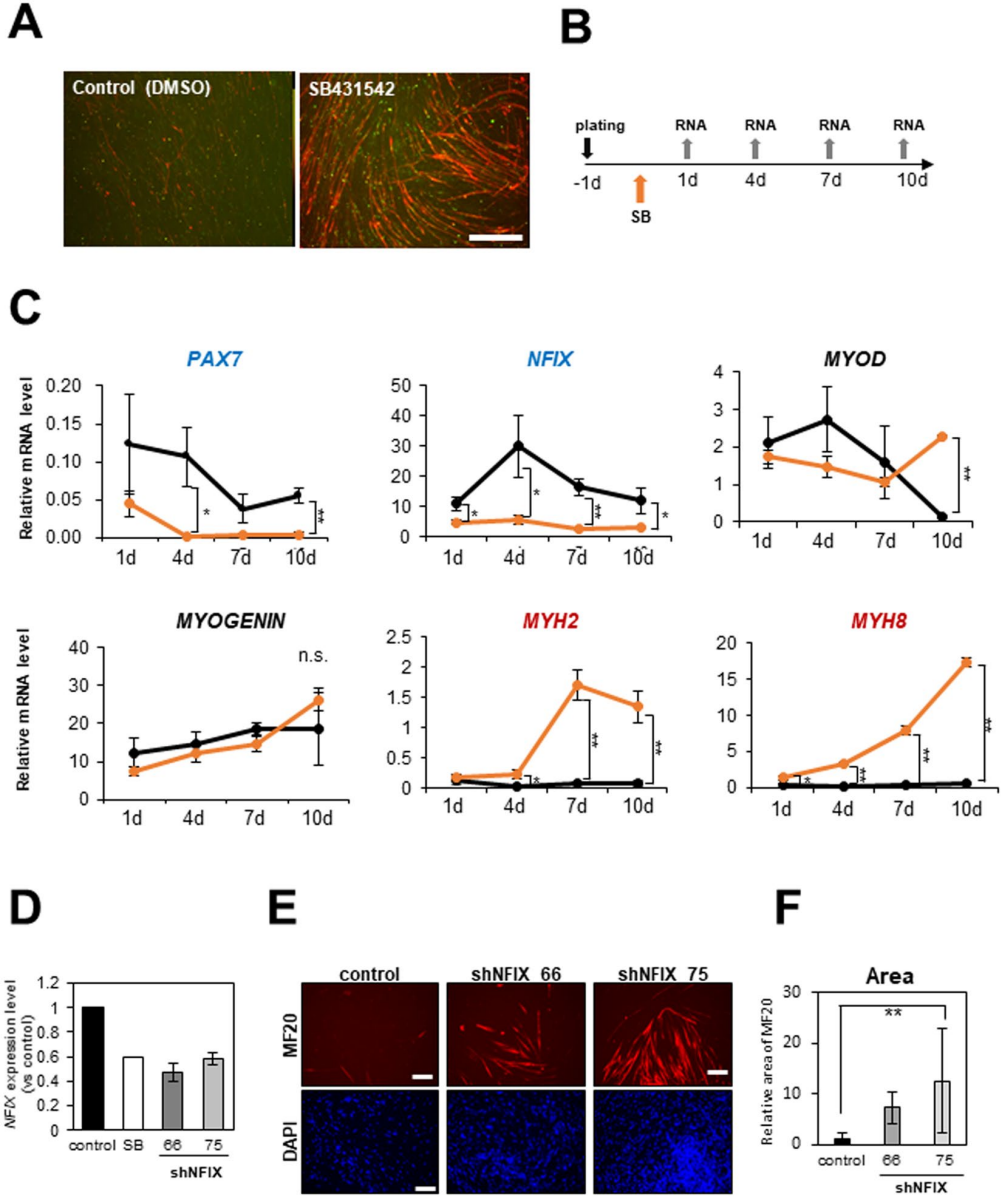
Gene expression of myogenic progenitors after SB341542 treatment. (**A**) Immunostaining of myotubes formed by 409B2 iPSC-derived CD271(+)CD57(−) myogenic progenitors after with MF20 (red), and MYOGENIN (green). After sorting, cells were replated onto collagen-coated plates and cultured for a week with or without SB431542 (10 μM). Scale bar: 500 μm. (**B**) Time schedules of SB administration and sampling. RNA was extracted 1, 4, 7, and 10 days after SB432542 treatment. (**C**) Gene expression examined by RT-qPCR. Average ± SD, *p < 0.05, **p < 0.01. (**D**) qPCR analysis to confirm the reduction of NFIX gene expression level by shRNAs. The expression level of NFIX was normalized to the control. Average ± SD. Control, SB (n = 1), shNFIX 66, 75 (n = 3). (**E**) 454E2 hiPSC-derived CD271(+)CD82(+)CD57(−) cells were transfected with shRNA plasmid targeting NFIX (66, 75) or control shRNA plasmid, selected with 1 μg/ml puromycin for 24 h, and cultured 10 d. The cells were then fixed, and stained with MHC antibody (MF20) and DAPI (Blue). Scale bar: 200 μm. (**F**) Relative area of MF20 signals are shown. Average ± SD (n = 3). Tukey-Kramer, **p < 0.01.

As soon as 24 hours after SB431542 administration, *NFIX* was downregulated (Fig. 5). To know the role of NFIX, we knock-downed NFIX using shRNA plasmids. Two shRNAs effectively reduced the NFIX mRNA level (about 50–55% compared with the control). The reduction of *NFIX* expression was almost the same as that of SB431542 treatment in 454E2 iPSC-derived myogenic cells (Fig. 5D) and 201B7 iPSC-derived myogenic cells (data not shown). The knockdown of *NFIX* promoted myogenic differentiation of hiPSC-derived myogenic cells to some extent (Fig. 5E), further suggesting that *NFIX* plays a role in maintenance of the undifferentiated state of myogenic progenitors.

### hiPSC-derived myogenic cells efficiently fused with adult myoblasts

**Figure 6:**
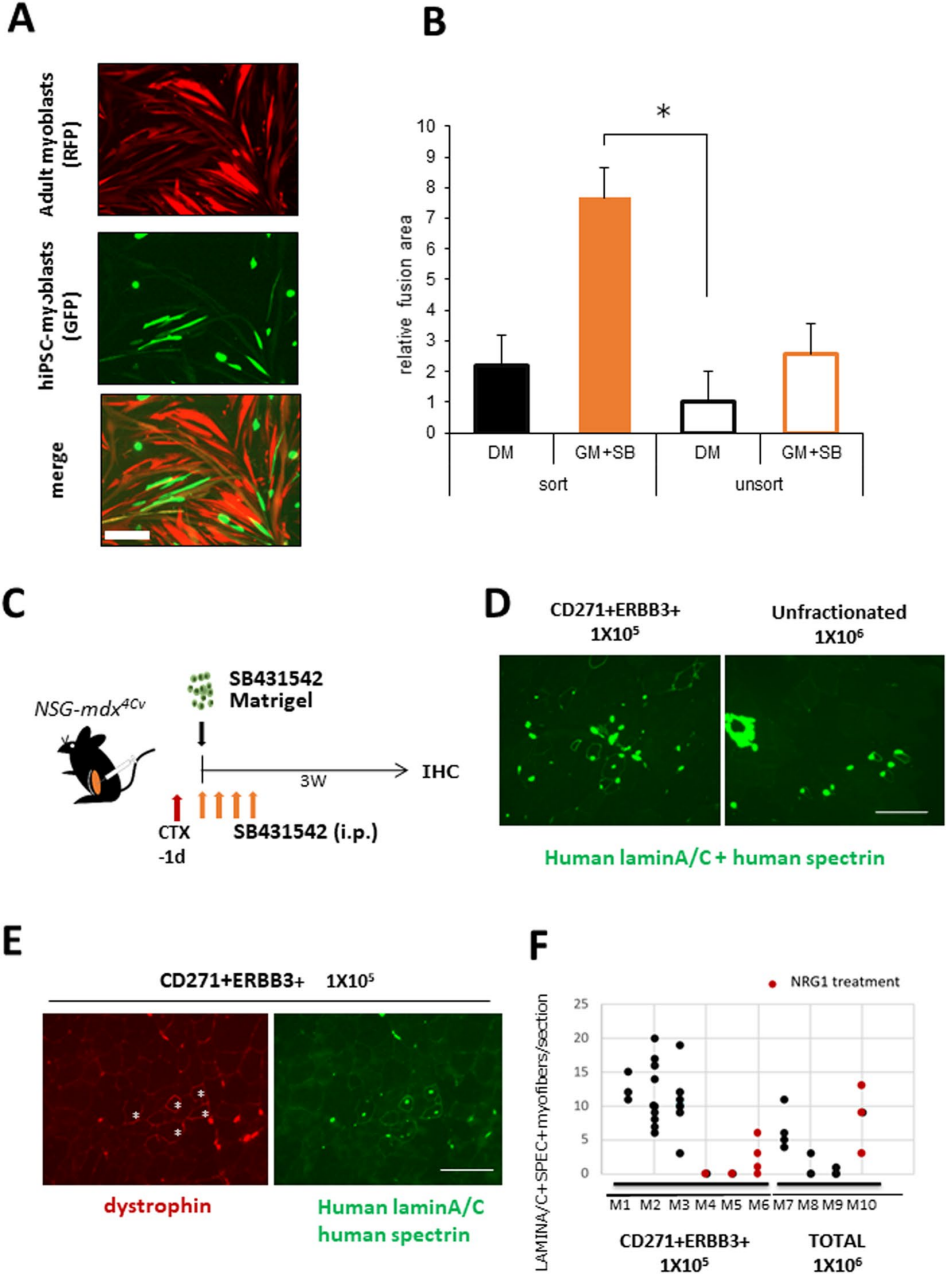
hiPSC-derived CD57(−) CD108(−) CD271(+) ERBB3(+) cells differentiated into myofibers in dystrophic TA muscle of *NSG-mdx*^4C*v*^ mice. (**A**) hiPSC-derived myogenic progenitors fused with adult myoblasts. hiPSC-derived CD57(−) CD108(−) CD271(+) ERBB3(+) myogenic cells (hiPSC-myoblasts) and unfractionated cells were labelled red by lentiviral transduction of pCLV-Ubic-TagRFP-IRES-Puro. Human myoblasts isolated from adult muscles were labelled green by a lentiviral vector, pCLV-CMV-copGFP-IRES-Neo. Scale bar = 200 µm. (**B**) Sorted (1 × 10^4^) and unsorted 201B7-derived myogenic cells (1 × 10^4^) were co-cultured with adult myoblasts (1 × 10^4^) in 48 well-plates for 4 days, and fusion was evaluated by measurement of yellow area using ImageJ. DM: 2% horse serum/DMEM. GM + SB: 10% FBS/DMEM + 10 µM SB431542. n = 3/group. *: < 0.05. (**C**) Experimental design of cell transplantation. Cells suspended in 10% Matrigel/LIF/IGF-1/PBS(−) solution, were directly injected into TA muscle using a 29 G needle. SB431542 was daily administered (i.p) to *NSG-mdx* mice (male, 3–6 months old) for four consecutive days. (**D**) Human spectrin-positive, human lamin A/C-positive myofibers in *NSG-mdx*^4C*v*^ after transplantation of CD57(−) CD108(−) CCD271(+) ERBB3(+) hiPSC (201B7)-derived myogenic cells. The injected muscles were analyzed three weeks after transplantation of 1 × 10^5^ CD57(−) CD108(−) CD271(+) ERBB3(+) cells (left) or 1 × 10^6^ unfractionated cells. (**E**) Immunostaining of section with dystrophin antibody after transplantation of CD57(−) CD108(−) CD271(+) ERBB3(+) cells (1 × 10^5^). (**F**) Numbers of human spectrin-positive, human lamin A/C-positive myofibers per transverse section. Each dot indicates the number of myofibers in a section. Red circles indicate pretreatment of the cells with recombinant Neuregulin1 (NRG1) for three days just before transplantation. NRG1 pretreatment did not improve the efficiency of transplantation. Bar indicates 200 μm in (**B**,**C**, and **D)**.

We next examined whether human iPSC-myogenic cells fuse with adult human myoblasts. To this end, we added hiPSC-derived myogenic cells (unfractionated and fractionated cells) labelled with copGFP to a differentiating culture of human adult myoblasts labelled with fluorescent TagRFP protein. Soon after the start of co-culture, the two kinds of myogenic cells started to fuse (Fig. 6A). The fusion efficiency of fractionated hiPSC-derived myogenic cells was higher than that of unfractionated cells (Fig. 6B). Importantly, SB431542 treatment again improved the fusion between human iPSC-myogenic cells fuse with adult human myoblasts (Fig. 6B).

### hiPSC-derived muscle progenitors regenerated myofibers and expressed dystrophin in TA muscles of *NSG-mdx*^*4Cv*^ mice

Next, we investigated whether hiPSC-derived CD57(−) CD108(−) CD271(+) ERBB3(+) cells are transplantable into TA muscles of *NSG-mdx*^4C*v*^ mice. We suspended the cells in PBS(−) containing 10% Matrigel and 10 μM SB431542. Based on the data reported by Hicks *et al*., we injected SB431542 daily into the peritoneal cavity of the recipient mice for four consecutive days to enhance differentiation of the transplanted cells^20^ (Fig. 6C). After transplantation of 1 × 10^5^ FACS-sorted cells, 12–13 human lamin A/C-positive (nuclear membrane) and human spectrin-positive (sarcolemma) myofibers were constantly detected on transverse sections; transplantation of 1 × 10^6^ unfractionated cells showed less efficiency (Fig. 6D,F). Human lamin A/C-positive, human spectrin-positive myofibers were also positive for dystrophin (Fig. 6E). Pretreatment of the cells with recombinant neuregulin1, a ligand of ERBB3, reduced the efficiency of transplantation of purified cells (Fig. 6F). This result suggests that ERBB3 signaling might suppress the differentiation of hiPSC-derived myogenic progenitors, although further analysis is needed to conclude this.

## Discussion

### Stable induction of myogenic progenitors from hiPS cells by new sphere method

To prepare a large quantity of myogenic cells from hiPS cells, we first tried to improve the EZ sphere method^21^ by continuous low-speed stirring of the culture using bioreactors. This method greatly increased cell yield, but the system was unstable and the average percentage of myogenic spheres of all experiments was not increased (Supplementary Figure 1). Therefore, to obtain myogenic cells more efficiently, we combined the EZ sphere method with a recently reported step-wise paraxial mesoderm induction protocol^18,19^. The first two or three steps of the directed differentiation induction using two small molecules (CHIR-99021 and LDN-193189) and three promyogenic growth factors (FGF-2, HGF, IGF-I) greatly increased the efficiency of sphere formation at the start of floating culture (Fig. 2B) and constantly induced PAX3+ PAX7+ premyogenic progenitors from the four hiPSC lines. Transfer of progenitors at the dermomyotomal or premyogenic stage in adhesion culture (i.e., protocol 3 or protocol 4 in Fig. 2) to floating culture showed high efficiency of muscle induction, although the protocol needed minor adjustments for each iPS clone (Fig. 2D). Sphere culture longer than six weeks reduced the percentage of myogenic spheres (Supplementary Figure 3). This is probably due to the faster growth of non-myogenic cells than differentiating muscle precursors at this stage.

### TGF-β signaling regulated differentiation of hiPSC-derived myogenic progenitors

Mouse embryonic myoblasts differentiate into myotubes in the presence of TGF-β. In contrast, differentiation of fetal myoblasts is strongly inhibited by TGF- β^24^. Recently, Hicks *et al*. reported that hPSC-derived myogenic progenitors express TGF- β signaling genes at higher levels than fetal myoblasts and that SB431542 induces the maturation of hPSC-derived skeletal muscle^20^. We observed that TGF-β inhibitors also promoted the differentiation of hiPSC-derived mononuclear myogenic progenitors, which otherwise form myotubes poorly in 10% FBS/DMEM medium. Interestingly, the blockage of TGF- β signaling promoted the differentiation of FACS-sorted, highly purified myogenic progenitors. Therefore, it is likely that muscle progenitors secrete TGF-β (or related factors) to regulate the timing of differentiation themselves. Gene expression analysis and knockdown experiments with *NFIX* shRNA suggested that inhibition of TGF- β signals promotes muscle differentiation of hiPSC-derived myogenic progenitors partly by reducing *NFIX* expression. Since a similar regulation is likely operating *in vivo*, the mechanisms by which *NFIX* inhibit differentiation of hiPSC-derived myogenic progenitors are of great interest and need to be elucidated.

### Human iPS cell-derived myogenic cells were transplantable

Transplantation experiments showed that myogenic cells derived from hiPSCs are transplantable and form dystrophin-positive myofibers. We also confirmed that hiPSC-derived myogenic cells efficiently fused with adult myoblasts *in vitro*. These data suggest that myogenic cells derived from hiPSCs using the sphere-based method are a good candidate cell for cell therapy of DMD. However, transplanted cells differentiated into host myofibers with low efficiency (10–15 myofibers/section with 1 × 10^5^ myogenic progenitors). In a recent report, Hicks *et al*. injected 1 × 10^6^ cells/TA muscle (ten times our cell numbers) and administered SB431542 directly into skeletal muscle every three days for two weeks^20^. Transplantation of larger numbers of the cells and direct SB431542 administration into muscle for a longer period might improve the efficiency of engraftment of our hiPSC-derived myogenic cells. The factors inhibiting the engraftment of transplanted cells and their fusion with host dystrophic myofibers, other than the TGF-β signal, also should be investigated in future studies.

## Experimental Procedures

### Ethical statement

Informed consent was obtained for all human iPS cells used in this study. Samples were anonymized upon leaving the clinic. The study was approved by the Ethics Committee of the National Center of Neurology and Psychiatry, and all methods were carried out in accordance with the guidelines.

### Cells

Human iPS cells (253G4, 201B7, 409B2, and 454E2) cultures established from healthy donors were provided from S. Yamanaka at the Center for iPS Cell Research and Application (CiRA), Kyoto University. Lines 253G4 and 201B7 were generated using retroviral vectors^6,26^, and 409B2 and 454E2 were generated using episomal vectors and proven to be integration-free^27^. 201B7-Myf5-tdTomato is a subline of 201B7 expressing tdTomato under Myf5 regulatory elements established by H. Sakurai at Kyoto University (Sakurai *et al*., unpublished data). Disease-specific iPS cells (GFPT1 #3 and #8) or DOX7 were established in our lab from fibroblasts of congenital myasthenic syndrome (CMS) patients. Ullrich-iPSCs were induced in our lab from fibroblasts carrying heterozygous mutations in the *COL6A2* gene (GM23778) obtained from the Coriell Institute.

Human iPS cells were cultured with mitomycin-C-inactivated mouse embryonic fibroblasts (MEF) on gelatin-coated dishes (Iwaki) in primate ES cell medium (ReproCELL) supplemented with 4 ng/ml FGF-2 (PEPROTECH). For feeder-free culture, human iPS cells were cultured on iMatrix-511 (Nippi)-coated 6-well plates in mTeSR1 medium (Stem Cell Technologies). Penicillin/streptomycin/amphotericin B (PSA, 1% v/v) (Wako) were added to the medium.

Primary myoblasts were purchased from LONZA (CC-2580, Lot 0000424745: 19-year-old, male, Black, and Lot 0000429275: 35-year-old, female, Caucasian) and from ThermoFisher (A11440, Lot 1669083: male, Caucasian, the age of the donor was not provided).

### Paraxial mesoderm induction

Paraxial mesoderm was induced from human iPS cells as described^18,19^. Human iPS cells were dissociated with TrypLE™ Select (Gibco) and plated as single cells per well on iMatrix-coated 6-well plates in mTeSR1 supplemented with 10 μM ROCK inhibitor (Y-27632, Wako) for 1 d. The medium was then changed to DMEM/F12 supplemented with 1% v/v Insulin-Transferrin-Selenium (ITS, Gibco), 3 μM CHIR-99021 (Tocris), and 0.5 μM LDN-193189 (stemgent) (CL medium). At day 3, 20 ng/ml FGF-2 (Pepro Tech) was added (CLF medium) for an additional 3 d. After 6 d of differentiation, the medium was changed to DMEM/F12 supplemented with 10 ng/mL HGF (Pepro Tech), 2 ng/mL IGF-1 (Pepro Tech), 20 ng/mL FGF-2 (Pepro Tech), and 0.5 μM LDN-193189 (HIFL medium) for 2 d. After 8 d differentiation, cells were cultured in DMEM/F12, 15% KSR (gibco), supplemented with 2 ng/mL IGF-1 for 4 d.

### Sphere culture

EZ sphere culture was performed as described previously^21^. Cells were cultured in Stemline for neural stem cells (S-3194, SIGMA Aldrich) supplemented with 100 ng/ml FGF-2 (Pepro Tech), 100 ng/ml EGF (Pepro Tech), and 5 µg/ml heparin sodium salt for 6–10 wk. Spheres were weekly chopped into 200 µm cubes by a McIlwain tissue chopper (Mickle Laboratory Engineering). The medium was replenished every 2–3 d. For stirring culture, spheres were cultured in the same medium in a 30-ml vessel with agitation at 55 rpm using a delta-shaped paddle impeller and a magnetic stirrer (Able Corp.), and chopped weekly into 200 μm-cubes.

### Muscle differentiation

After floating culture, spheres were plated on collagen-coated 24-well plates (Iwaki) at one sphere per well and induced to differentiate into myotubes in DMEM containing 10% fetal bovine serum (FBS) (Gibco) up to 4 wk. DMEM supplemented with 10% FBS medium supported cell growth and after reaching confluence, cells started to fuse to form multinucleated myotubes. For FACS, the spheres were plated onto 100-mm dishes for a week.

### TGF-β or Notch signal inhibition

Cells were plated on collagen-coated 12- or 24-well plates and cultured in DMEM containing 10% FBS, 10 μM SB431542 (Wako), and/or 10 μM DAPT (Wako). The medium was changed every other day up to 4 wk.

### NFIX siRNA

hiPSC-derived CD271(+)CD82(+)CD57(−) cells (5 × 10^4^ cells/well in 24-well plates) were transfected with MISSION^®^ pLKO.1-puro non-mammalian shRNA control plasmids or NFIX MISSION shRNA plasmids (14775, 234766) (Sigma-Aldrich) using FuGENE HD transfection reagent according to the manufacturer’s protocol (Promega). Transduced cells were selected with 1 μg/ml puromycin (Calbiochem) and cultured for ten days in 10%FCS/DMEM on collagen-coated 24-well plates. MF20 signals in more than eight fields were quantified with a KEYENCE BZ-X Analyzer and hybrid cell count software.

### Immunocytochemistry

Myogenic differentiation was evaluated with immunocytostaining for myosin heavy chain (MF20, R&D Systems), MYOGENIN (Santa Cruz Biotechnology, FD5 or rabbit polyclonal), Pax7 (Santa Cruz Biotechnology, PAX7), and MyoD (5.8 A or rabbit polyclonal; Santa Cruz Biotechnology). Cells were fixed in 4% paraformaldehyde and then permeated with 0.1% Triton-X100 for 10 min at room temperature. After blocking with 5% goat serum/2% bovine serum albumin in PBS(−), cells were incubated with primary antibodies (100–400 dilution) overnight at 4 °C. The next day, the cells were washed in PBS(−) and incubated with fluorescence-labeled secondary antibodies (Alexa 568-labeled goat-anti-mouse IgG2b or Alexa488-labeled goat anti-rabbit IgG)(Molecular Probes) for 2 h. After washing with PBS(−), the nuclei were stained with Hoechst 33258 (Dojindo). The images were recorded using a KEYENCE BZX-710 and analyzed with hybrid cell count software (Keyence Corp.) or IX71 (Olympus) equipped with an ORCA-R^2^ digital CCD camera and AQUACOSMOS2.6 software (Hamamatsu Photonics).

### RNA isolation, cDNA synthesis, and qPCR array

Total RNA was isolated from cells with Trizol (Invitrogen) or an RNeasy Mini Kit (Qiagen), reverse-transcribed into cDNA using PrimeScript RT reagent kit (Perfect Real Time, Takara), and amplified by primer sets (Supplementary Table 2) and SYBR Premix EX Taq II (Til RNaseH Plus, Takara). SYBER green signals were monitored by a CFX Connect system (Bio-Rad), and ΔCt (1/2^(Cq of the gene-median Cq)) was calculated.

### FACS analysis and cell sorting

Cells were incubated with antibodies in 200 μl PBS containing 2% FBS (wash buffer) at the dilutions suggested by suppliers for 30 min on ice. Then the cells were washed in wash buffer and analyzed and sorted using a BD FACSAria Fusion cell sorter (BD Biosciences). The following antibodies were used: CD29-PE (clone TS2/16, eBioscience), CD45-PE (clone HI30, BD Pharmingen), CD56-FITC (clone B159, BD Pharmingen), CD57(HNK-1)-PE (clone TB03, Miltenyi Biotec), CD82-APC (clone REA211, Miltenyi Biotec), ERBB3-APC (clone REA508, Miltenyi Biotec), CD105-FITC (clone 266, BD Pharmingen), CD108-PE (clone KS-2, BD Pharmingen), CD140a (PDGFRα)–PE (clone αR1, BD Pharmingen), CD146/MCAM -PE (clone P1H12, BD Pharmingen), CD271-PE (clone C40-1457, BD Pharmingen), CD271-BD Horizon BB515 (BD), CD318-APC (clone REA194, Miltenyi Biotec), CD325/N-cadherin-PE (clone 8C11, BioLegend), C-MET-APC (clone 95106, R&D systems), SSEA4-PE (clone MC813-70, BD Pharmingen), TRA-1-60-Alexa 488 (clone TRA-1-60, BD Pharmingen), and TRA-1-81-Alexa647 (clone TRA-1-81, BD Pharmingen). After sorting, cells were plated on collagen-coated 12- or 48-well plates and cultured up to 10 d.

### Mice

Immunodeficient muscular dystrophy model mice (*NSG-mdx*^4c*v*^ mice)^28^ were provided by M. Kyba at the University of Minnesota. Mice were allowed ad libitum access to food and drinking water. The experimental Animal Care and Use Committee of the National Institute of Neuroscience, National Center of Neurology and Psychiatry (NCNP), Japan, approved all experimental protocols in this study. All experiments were performed in accordance with the guidelines and regulations.

### Cell transplantation

One day prior to transplantation, tibialis anterior (TA) muscles were injured by injection of cardiotoxin (CTX) (100 μl of 10 mM). Cells were dissociated with 0.05% Trypsin, filtered through 40 μm mesh (Falcon), and resuspended in PBS(−) with 10% Matrigel (BD Bioscience), 10 µM SB431542 (Wako), 100 ng/mL LIF (Prospec)^29^, 100 ng/mL HGF (Peprotec), and fluorescent beads (Life Technologies). Sixty µL of a cell preparation containing 100,000 to 1,000,000 cells was injected in TA muscles of 2- to 3-month-old *NSG-mdx*^4c*v*^ male mice three separate times (20 µL per injection with 30 min intervals). Injection was done under general anesthesia. After 1 month, TA muscles were collected and processed for cryosectioning (6–10 µm thick) and immunohistochemistry.

### Immunohistochemistry

Muscle tissue sections were fixed for 10 min in cold acetone and then air-dried for more than 30 min. After blocking with 5% goat serum/2% bovine serum albumin in PBS(−), cells were incubated with primary antibodies: human lamin A/C (Santa Cruz), human spectrin (Leica), or dystrophin (Abcam) (100–400 dilution) overnight at 4 °C. The next day, sections were washed in PBS(−) and incubated with fluorescence-labeled secondary antibodies (Alexa 488-labelled goat-anti-mouse IgG2b, or Alexa568-labelled goat anti-rabbit IgG)(Molecular Probes) for 2 h. Sections were mounted in Vecta Shield with DAPI (Vector).

### Statistics

The significance of differences among experimental groups was assessed by Student’s t test or one-way ANOVA followed by Tukey’s hoc test.

## Electronic supplementary material

Supplementary Information

## References

[1] Partridge TA, Morgan JE, Coulton GR, Hoffman EP, Kunkel LM (1989). Conversion of mdx myofibres from dystrophin-negative to -positive by injection of normal myoblasts. Nature.

[2] Mouly V (2005). Myoblast transfer therapy: is there any light at the end of the tunnel?. Acta Myol..

[3] Negroni E (2015). Invited review: Stem cells and muscle diseases: advances in cell therapy strategies. Neuropathol. Appl. Neurobiol..

[4] Montarras D (2005). Direct isolation of satellite cells for skeletal muscle regeneration. Science.

[5] Ikemoto M (2007). Autologous transplantation of SM/C-2.6(+) satellite cells transduced with micro-dystrophin CS1 cDNA by lentiviral vector into mdx mice. Mol. Ther..

[6] Takahashi K (2007). Induction of pluripotent stem cells from adult human fibroblasts by defined factors. Cell.

[7] Kodaka Y, Rabu G, Asakura A (2017). 2017. Skeletal muscle cell induction from pluripotent stem cells. Stem Cells Int..

[8] Warren L (2010). Highly efficient reprogramming to pluripotency and directed differentiation of human cells with synthetic modified mRNA. Cell Stem Cell.

[9] Darabi R (2012). Human ES- and iPS-derived myogenic progenitors restore DYSTROPHIN and improve contractility upon transplantation in dystrophic mice. Cell Stem Cell.

[10] Abujarour R (2014). Myogenic differentiation of muscular dystrophy-specific induced pluripotent stem cells for use in drug discovery. Stem Cells Trans. Med..

[11] Maffioletti SM (2015). Efficient derivation and inducible differentiation of expandable skeletal myogenic cells from human ES and patient-specific iPS cells. Nat. Protoc..

[12] Goudenege S (2012). Myoblasts derived from normal hESCs and dystrophic hiPSCs efficiently fuse with existing muscle fibers following transplantation. Mol. Ther..

[13] Tanaka A (2013). Efficient and reproducible myogenic differentiation from human iPS cells: prospects for modeling Miyoshi Myopathy *in vitro*. PLoS One..

[14] Awaya T (2012). Selective development of myogenic mesenchymal cells from human embryonic and induced pluripotent stem cells. PLoS One..

[15] Xu C (2013). A zebrafish embryo culture system defines factors that promote vertebrate myogenesis across species. Cell.

[16] Borchin B, Chen J, Barberi T (2013). Derivation and FACS-mediated purification of PAX3 + /PAX7 + skeletal muscle precursors from human pluripotent stem cells. Stem Cell Rep..

[17] Shelton M (2014). Derivation and expansion of PAX7-positive muscle progenitors from human and mouse embryonic stem cells. Stem Cell Rep..

[18] Chal J (2015). Differentiation of pluripotent stem cells to muscle fiber to model Duchenne muscular dystrophy. Nat. Biotechnol..

[19] Chal J (2016). Generation of human muscle fibers and satellite-like cells from human pluripotent stem cells *in vitro*. Nat Protoc..

[20] Hicks MR (2018). ERBB3 and NGFR mark a distinct skeletal muscle progenitor cell in human development and hPSCs. Nat Cell Biol..

[21] Hosoyama T (2014). Derivation of myogenic progenitors directly from human pluripotent stem cells using a sphere-based culture. Stem Cells Transl. Med..

[22] Jiwlawet S (2017). Differentiation and sarcomere formation in skeletal myocytes directly prepared from human induced pluripotent stem cells using a sphere-based culture. Differentiation.

[23] Uezumi A (2016). Cell-Surface Protein Profiling Identifies Distinctive Markers of Progenitor Cells in Human Skeletal Muscle. Stem Cell Rep..

[24] Cusella-De A (1994). Differential response of embryonic and fetal myoblasts to TGF beta: a possible regulatory mechanism of skeletal muscle histogenesis. Development.

[25] Mourikis P (2012). A critical requirement for notch signaling in maintenance of the quiescent skeletal muscle stem cell state. Stem Cells.

[26] Nakagawa M (2008). Generation of induced pluripotent stem cells without Myc from mouse and human fibroblasts. Nat. Biotechnol..

[27] Okita K (2011). A more efficient method to generate integration-free human iPS cells. Nat. Methods.

[28] Arpke RW (2013). A new immuno-, dystrophin-deficient model, the NSG-mdx(4Cv) mouse, provides evidence for functional improvement following allogeneic satellite cell transplantation. Stem Cells.

[29] Ito N, Shimizu N, Tanaka H, Takeda S (2016). Enhancement of satellite cell transplantation efficiency by leukemia inhibitory factor. J. Neuromuscular Dis..

