# Supplementary material for: Premyogenic progenitors derived from human pluripotent stem cells expand in floating culture and differentiate into transplantable myogenic progenitors
Source: Sci Rep. 2018 Apr 26;8:6555. doi: 10.1038/s41598-018-24959-y (PMC5920060; doi:10.1038/s41598-018-24959-y)
Supplement: Supplementary file 1 — Supplementary Information [file 41598_2018_24959_MOESM1_ESM.pdf]

Research article

Premyogenic progenitors derived from human pluripotent stem cells expand in floating culture and differentiate into transplantable myogenic progenitors

Fusako Sakai-Takemura[1)], Asako Narita[1)], Satoru Masuda[1)], Toshifumi Wakamatsu[1)], Nobuharu Watanabe[1)], Takashi Nishiyama[1)], Ken'ichiro Nogami[1)], Matthias Blanc[1)], Shin'ichi Takeda[1)], Yuko Miyagoe-Suzuki[1)#]

Department of Molecular Therapy, National Institute of Neuroscience, National Center of Neurology and Psychiatry, Tokyo 187-8502, Japan

# corresponding author

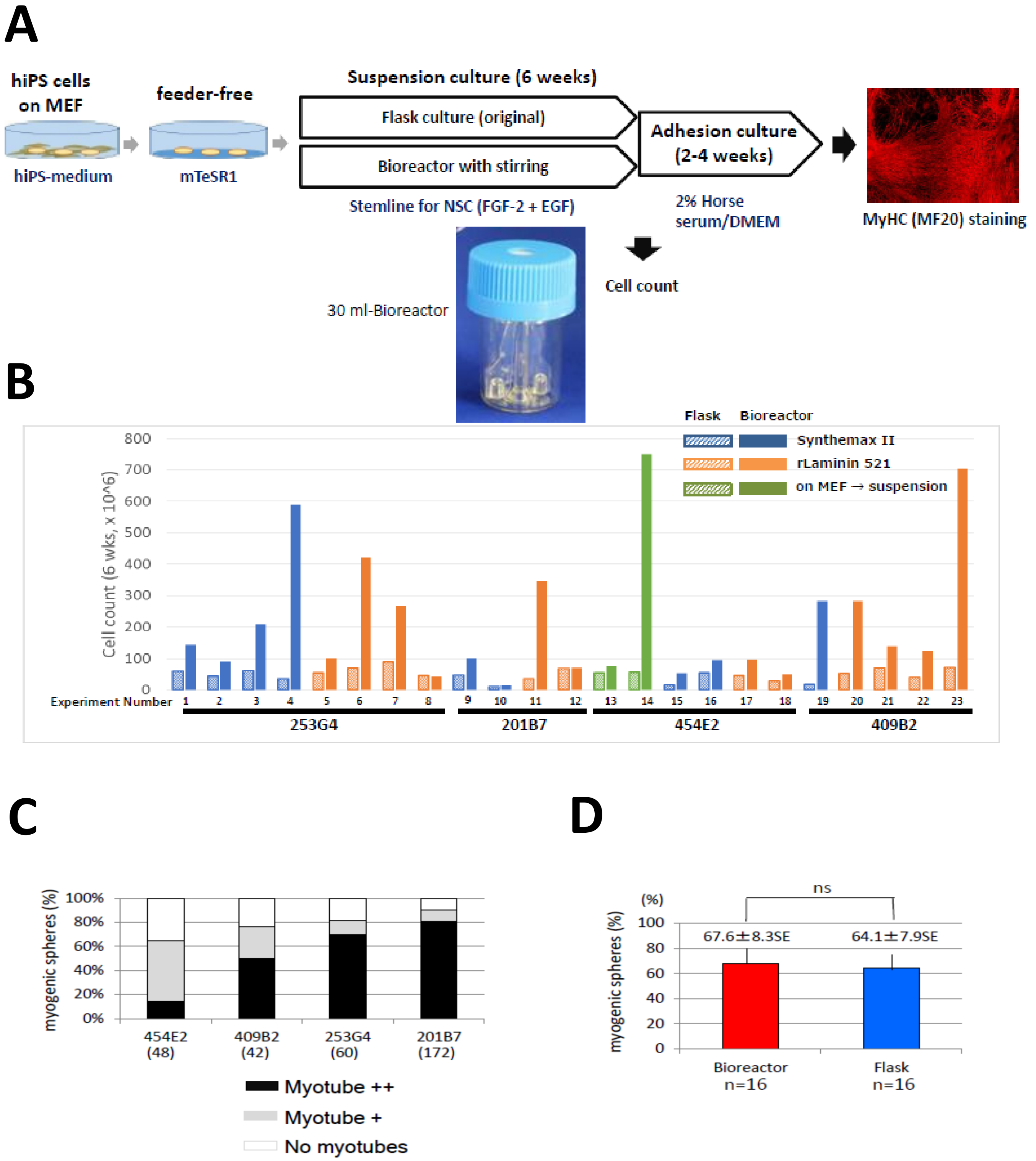
A
hiPS cells
on MEF
feeder-free
hiPS-medium
mTeSR1
Suspension culture (6 weeks)
Flask culture (original)
Bioreactor with stirring
Stemline for NSC (FGF-2 + EGF)
Adhesion culture
(2-4 weeks)
2% Horse
serum/DMEM
MyHC (MF20) staining
Cell count
30 ml-Bioreactor
B
Flask
Bioreactor
Synthemax II
rLaminin 521
on MEF → suspension
Cell count (6 wks, x 10^6)
800
700
600
500
400
300
200
100
0
Experiment Number
1 2 3 4 5 6 7 8 9 10 11 12 13 14 15 16 17 18 19 20 21 22 23
253G4
201B7
454E2
409B2
C
myogenic spheres (%)
100%
80%
60%
40%
20%
0%
454E2
(48)
409B2
(42)
253G4
(60)
201B7
(172)
Myotube ++
Myotube +
No myotubes
D
ns
(%)
myogenic spheres (%)
100
80
60
40
20
0
67.6±8.3SE
64.1±7.9SE
Bioreactor
n=16
Flask
n=16

Supplementary Figure 1

Continuously stirred sphere culture supported cell growth of hiPSC-derived spheres well and promoted their differentiation into skeletal muscle lineage

Experimental design. Human iPS cells cultured on mitomycin C-treated mouse embryonic fibroblasts (MMC-MEFs) were transferred to SynthemaxII (corning)- or laminin521(BioLamina)-coated dishes and cultured in mTeSR1. After reaching 80–90% confluency, hiPS cells were collected using a cell scraper, and transferred to ultra-low adhesion T75 flasks or constantly stirred 30-ml bioreactors and cultured for 6 weeks. Then spheres were plated on collagen-coated dishes in 2% horse serum (HS)/DMEM medium.

Numbers of cells obtained by stirred floating culture (filled) or by the original EZ sphere culture (striped) for 6 weeks were compared. Results of four human iPSC clones (253G4, 201B7, 454E2, and 409B2) (23 pairs of experiments) are shown. Blue indicates feeder-free culture of iPS cells on SynthemaxII. Orange indicates feeder-free culture of iPS cells on recombinant laminin521 (rLaminin521). Green indicates that iPS cells were directly transferred from co-culture with MEF to suspension culture.

Percentage of myogenic spheres of four human iPS cell clones. Each human iPS clone had different myogenic activities. Numbers in () are counted wells.

Percentages of myogenic spheres cultured in bioreactors (red) or in flasks (blue). n.s.: not significant. Student's *t* test.

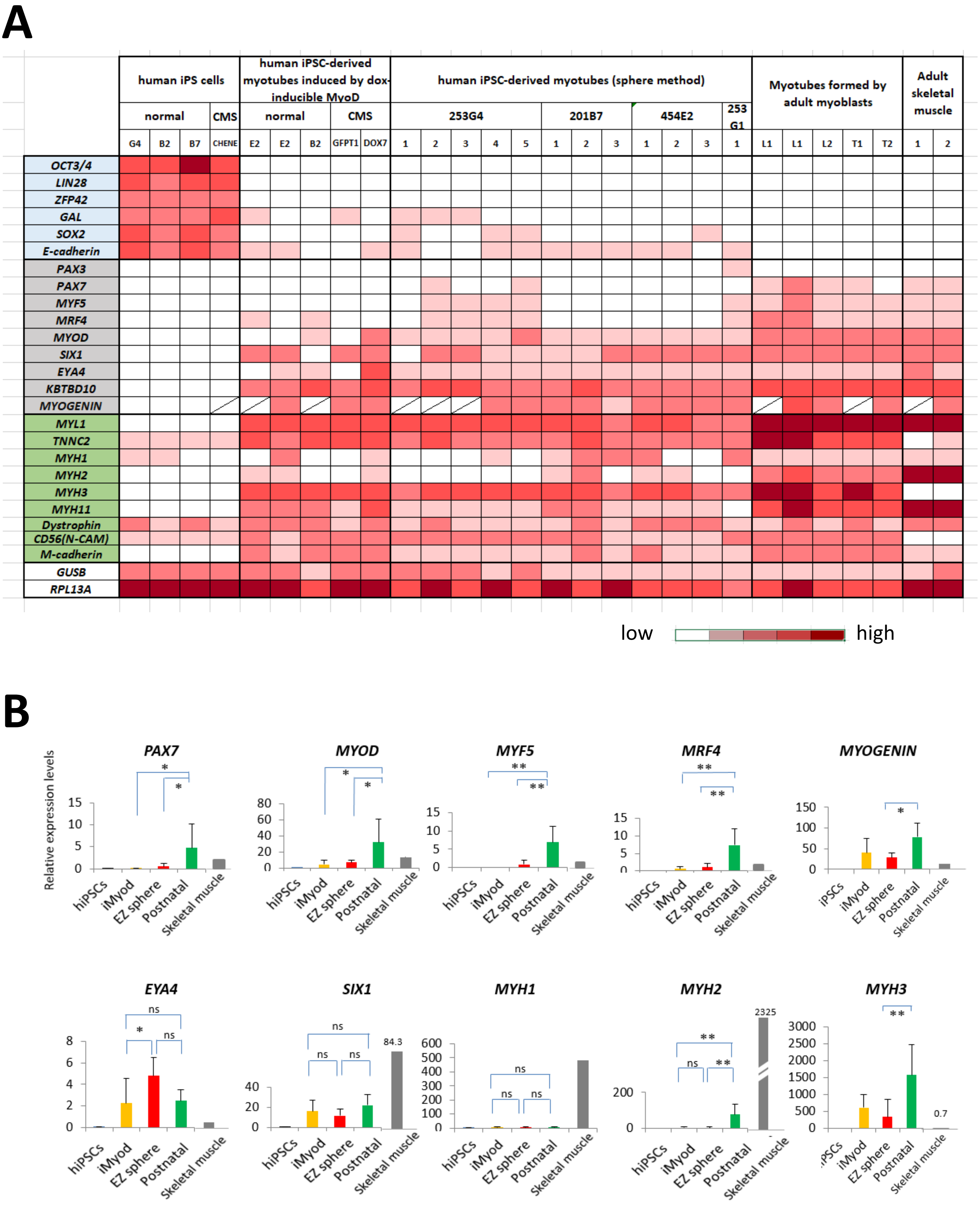
A
human iPS cells
human iPSC-derived myotubes induced by dox-inducible MyoD
human iPSC-derived myotubes (sphere method)
Myotubes formed by adult myoblasts
Adult skeletal muscle
normal
CMS
normal
CMS
253G4
201B7
454E2
253 G1
G4 B2 B7 CHENE E2 E2 B2 GFPT1 DOX7 1 2 3 4 5 1 2 3 1 2 3 1 L1 L1 L2 T1 T2 1 2
OCT3/4
LIN28
ZFP42
GAL
SOX2
E-cadherin
PAX3
PAX7
MYF5
MRF4
MYOD
SIX1
EYA4
KBTBD10
MYOGENIN
MYL1
TNNC2
MYH1
MYH2
MYH3
MYH11
Dystrophin
CD56(N-CAM)
M-cadherin
GUSB
RPL13A
low
high
B
Relative expression levels
PAX7
MYOD
MYF5
MRF4
MYOGENIN
EYA4
SIX1
MYH1
MYH2
MYH3
hiPSCs
iMyod
EZ sphere
Postnatal
Skeletal muscle
ns
84.3
2325
0.7

- **Supplementary Figure2**

Gene expression of parental hiPSCs, lentivirally transduced MyoD-induced myotubes, myotubes induced by EZ-sphere method, myotubes formed by adult myoblasts, and adult skeletal muscle tissue

RNA was extracted from parental human iPS cells, human iPSC-derived myotubes induced by dox-inducible MyoD[30], and human iPSC-derived myotubes induced by sphere method, and analyzed by RT-qPCR. Relative expression levels are shown by gradation of red color. hiPS cells derived from patients with congenital myasthenic ***syndrome*** caused by a *GTPT1* mutation(GFPT1), *CHENE* mutation (CHENE) or *DOX7* mutation were included in the analysis. Data of myotubes formed by adult myoblasts and adult skeletal muscle were also shown. Diagonal lines indicate the data deleted from the analysis due to multiple amplified bands.

Statistical analysis of the expression levels of *EYA4*, *SIX1*, *PAX7*, *MYF5*, *MRF4*, and *MYOD* in a. iPSCs: human iPS cells (n=4), iMyoD: inducible MYOD-induced myotubes (n=5), EZ sphere: single sphere-derived myotubes (n=11), adult myotubes: myotubes formed by cell fusion of purchased adult myoblasts (n=5, from 3 samples). Data are shown as average ± SD. *, $p<0.05$, **, $p<0.01$. ns: not significant. Tukey-Kramer. There was a significant difference between hiPSCs and myotubes in each comparison, but the differences are not indicated in the graph to avoid confusion. The data of adult skeletal muscle (gray) were not included in the statistical analysis.

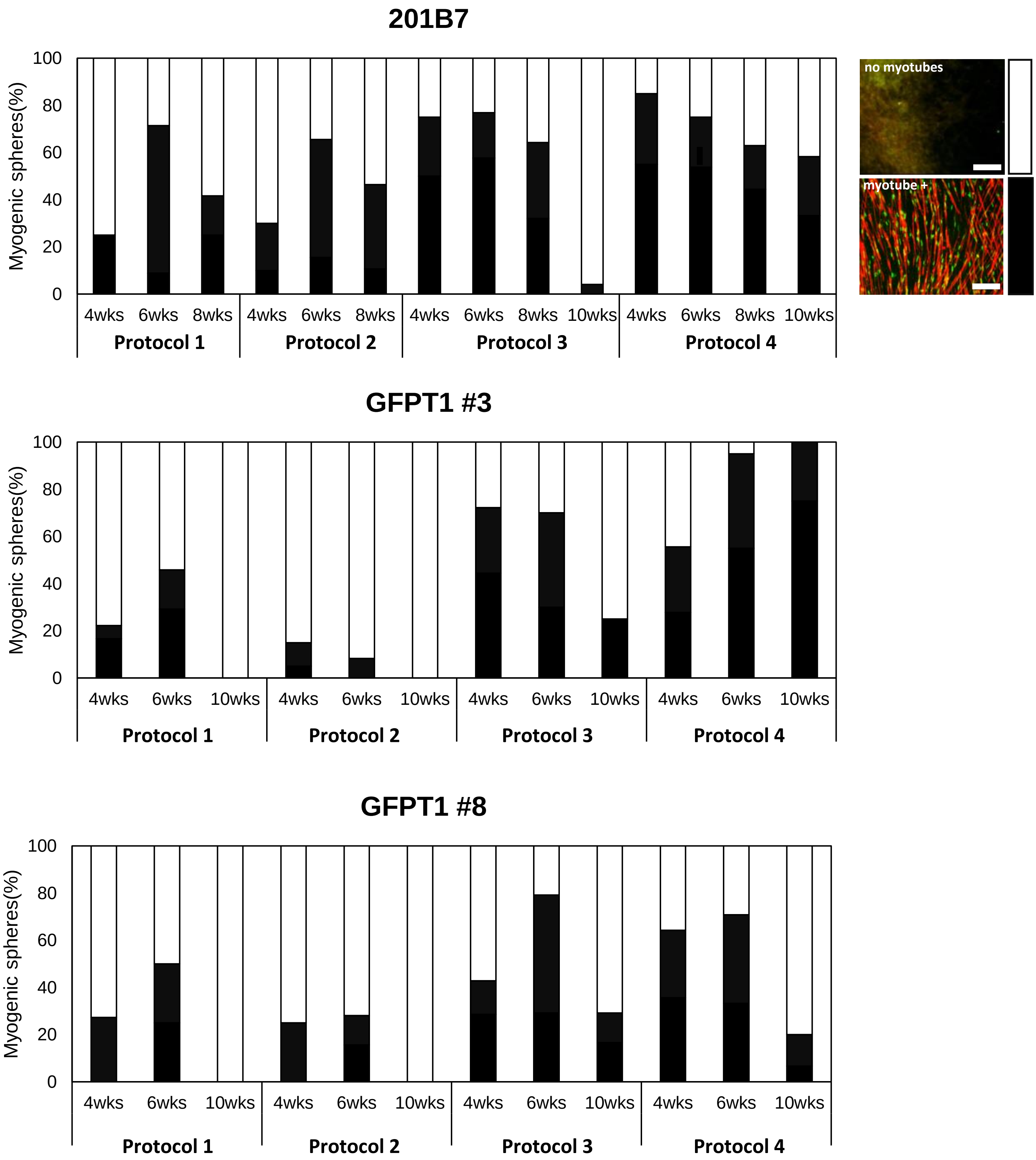
201B7
Myogenic spheres(%)
100
80
60
40
20
0
4wks
6wks
8wks
10wks
Protocol 1
Protocol 2
Protocol 3
Protocol 4
no myotubes
myotube +
GFPT1 #3
Myogenic spheres(%)
4wks
6wks
10wks
Protocol 1
Protocol 2
Protocol 3
Protocol 4
GFPT1 #8
Myogenic spheres(%)
4wks
6wks
10wks
Protocol 1
Protocol 2
Protocol 3
Protocol 4

Supplementary Figure3

Floating culture longer than six weeks reduced myogenic activity

201B7, GFPT1 #3, and GFPT1 #8 iPSCs were induced to differentiate into a skeletal muscle lineage as shown in Figure 2A, cultured as spheres for 4 weeks, 6 weeks, 8 weeks, or 10 weeks, and then plated onto collagen-coated 24-well plates (1 sphere/well). Floating culture longer than 6 weeks tended to reduce the percentage of myogenic spheres, except for GFPT1 #3.

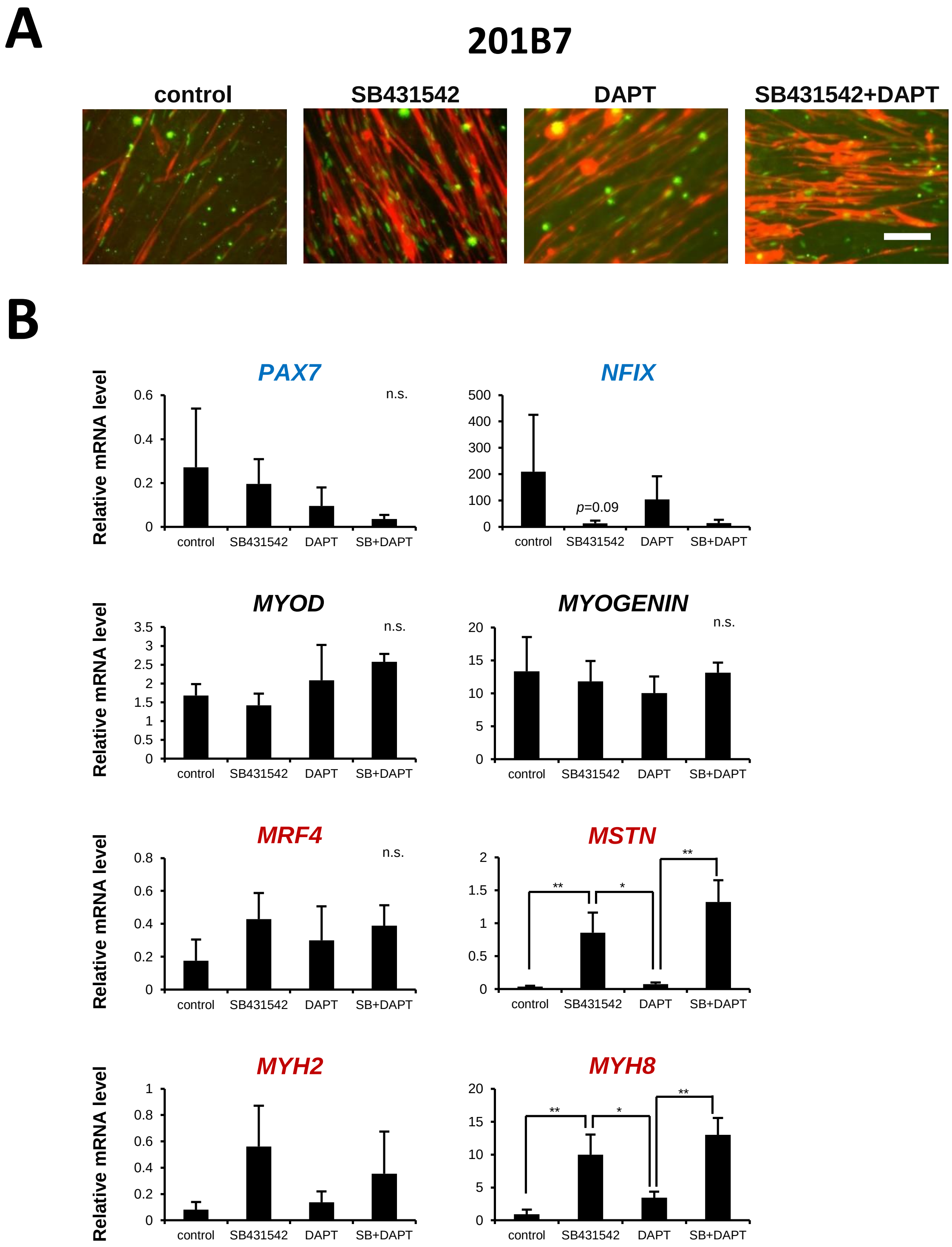

A
201B7
control
SB431542
DAPT
SB431542+DAPT
B
PAX7
NFIX
MYOD
MYOGENIN
MRF4
MSTN
MYH2
MYH8
Relative mRNA level
n.s.
p=0.09
**
*
control
SB431542
DAPT
SB+DAPT

Supplementary Figure 4 (related to Figure 4)

Effects of SB431452 and DAPT1 on differentiation of 201B7 hiPSC-derived myogenic cells

After 6-week muscle induction and 4-week adhesion culture, 201B7 iPS cells were cultured in the presence of SB431542 (TGF-β inhibitor), DAPT (Notch inhibitor), or both (SB431542+DAPT). Ten days after administration, muscle differentiation was evaluated with MF20 (red), and MYOGENIN (green). Scale bar = 100 µm.

RT-qPCR analysis of myogenic regulators and muscle-specific genes in control, SB431542-, DAPT-, and SB431542+DAPT-treated myogenic cells derived from 201B7 iPSCs. **: $p<0.01$, *: $p<0.05$, n.s. (n=3).

Supplementary Table 1

Summary of the efficiency of muscle induction from control or patient hiPSCs using the original EZ sphere and the new protocol

hiPSC lines	Number of experiment s	Induction methods	myogenic spheres (%) (myogenic spheres / total spheres)	Average (%) (SD)
201B7	4	original EZ sphere	97.2 (35/36)<br>100 (12/12)<br>58.3 (7/12)<br>91.7 (11/12)	86.8 (19.3)
201B7	3	new method (protocol 3)	100 (12/12)<br>100 (12/12)<br>83.3 (10/12)	94.4 (9.6)
409B2	4	original EZ sphere	12.5 (2/16)<br>75.0 (9/12)<br>83.3 (10/12)<br>100 (12/12)	67.7 (37.2)
409B2	4	new method (protocol 2+3)	100 (12/12)<br>95.8 (23/24)<br>83.3 (10/12)<br>91.7 (22/24)	96.9 (3.4)
COL6A2-iPSC (Ullrich congenital muscular dystrophy)	6 lines x1	original EZ sphere	33.3 (4/12)<br>7.7 (1/13)<br>69.6 (16/23)<br>76.2 (16/21)<br>29.2 (7/24)<br>58.3 (14/24)	45.7 (24.3)
GFPT1#3 (CMS)	3	new method (protocol 4)	91.7 (11/12)<br>100 (8/8)<br>75.0 (6/8)	88.9 (12.7)
GFPT1#8 (CMS)	3	new method (protocol 4)	75.0 (6/8)<br>37.5 (3/8)<br>75.0 (6/8)	62.5 (21.7)

Supplementary Table 2

Sequences of qPCR primers used in this study

Gene	Forward primer (5'-3')	Reverse primer (5'-3')	Product Size (bp)	GenBank No.	Primer set ID
OCT3/4	GACAGGGGGAGGGGAGGAGCTAGG	CTTCCCTCCAACCAGTTGCCCCAAAC	144	NM_001173531.2	ref. 6
NANOG	CCTGTGATTTGTGGGCCTGA	CTCTGCAGAAGTGGGTTGTTTG	168	NM_001297698.1	HA237725**
SOX2	CCAAGATGCACAACTCGGAGA	CCGGTATTTATAATCCGGGTGCT	143	NM_003106.3	HA173580
T	ACCAATGAGATGATCGTGACCAA	GCCAGACACGTTCACCTTCAG	69	NM_001270484.1	HA214597
TBX6	GAGACCACATTCATCTCCGTGAC	ATAGGCCTCTGTGGCTGCTG	177	NM_004608.3	HA128466
PAX3	CCATACGTCCTGGTGCCATC	TGAACATGCCCGGGTTCTC	105	NM_181458.3	HA146477
PAX7	GGCGACTCCGGATGTAGAGA	AGCACGCGGCTAATCGAAC	153	NM_002584.2	HA176960
MYOD1	AAATCAGGCCGGACAGGAG	ATAGCAAAGTGCTGGCAGTCTGAA	72	NM_002478.4	HA132734
MYOG	not disclosed				QT00001722*
MYF5	ACTACTATAGCCTGCCGGGACA	TGTTGGATAAGCAATCCAAGCTG	198	NM_005593.2	HA041385
MRF4	not disclosed				QT00201929*
NFIX	GTCTGGAATGTGACGGAGCTG	AGGGTCACCTGGTTGATGTTGTAG	122	NM_001271044.2	HA222201
MSTN	CGGAAACAATCATTACCATGCCTAC	TCTCGACGGGTCTCAAATATATCCA	150	NM_005259.2	HA293620
ERBB3	ACCAGACACTGTACAAGCTCTACGA	ACGAGGACATAGCCTGTCACTTC	130	NM_001005915.1	HA264089
ACTA1	CGAGCCGAGAGTAGCAGTTGTAG	AGCCATTGTCGCACACGAG	93	NM_001100.3	HA127293
TTN	TGCTGTGCACATCCAACCTG	TCGGCGGCCACTACTACCTTA	84	NM_133379.4	HA293054
MYH1	AATGTCCAAGGCCAACAGTGA	AGCATCCTGCAGACGCTGA	121	NM_005963.3	HA149993
MYH2	AAGGTCTGCGCAAACATGAGAG	GCTGGAGCTTGCGGAATTTAG	189	NM_001100112.1	HA133457
MYH3	ATGAAGCCATCCGGCTCAAG	CATCCAGGTGGAGCTGCGTA	150	NM_002470.3	HA114500
MYH8	GGAGCAAGCTGAGCCAGATG	CACAGTCTGGCCTTTGGTGA	142	NM_002472.2	HA144814
TNNC1	GCCAGCATGGATGACATCTACAA	CAGCACGAAGATGTCGAAGG	93	NM_003280.2	HA130406
DMD	TATGACCGCCTGGAGCAAGA	TTCAGCAGCCAGTTCAGACACA	80	NM_000109.3	HA084845
GUSB	AAACGATTGCAGGGTTTCAC	CTCTCGTCGGTGACTGTTCA	171	NM_000181	BIOMOL
GAPDH	GCACCGTCAAGGCTGAGAAC	TGGTGAAGACGCCAGTGGA	138	NM_002046.5	HA067812

*: QTnnn: Qiagen primer
** HA-nnnn: Takara primers
